# *In-Vitro* Anticancer Evaluation of Some Novel Thioureido-Benzensulfonamide Derivatives

**DOI:** 10.3390/molecules21040409

**Published:** 2016-03-25

**Authors:** Mostafa M. Ghorab, Mansour S. Alsaid, Mohammed S. Al-Dosari, Marwa G. El-Gazzar, Ahmed H. Arbab

**Affiliations:** 1 Department of Pharmacognosy, College of Pharmacy, King Saud University, P. O. Box 2457, Riyadh 11451, Saudi Arabia; msalsaid@ksu.edu.sa (M.S.A.); msdosari@yahoo.com (M.S.A.-D.); arbabssn@gmail.com (A.H.A.); 2 Department of Drug Radiation Research, National Center for Radiation Research and Technology (NCRRT), Egyptian Atomic Energy Authority (EAEA), Nasr City, Cairo 113701, Egypt; marwagalalgazzar@yahoo.com

**Keywords:** synthesis, sulfonamides, thioureido, anticancer activity

## Abstract

A novel series of sulfonamide derivatives (14 compounds) bearing thiourea moieties were efficiently synthesized and evaluated for their possible *in vitro* anticancer activity against four human tumor cell lines. The results indicated that compound **6** was the most potent, showing effectiveness on all the tested cell lines. Compounds **7** and **10** also showed promising results.

## 1. Introduction

Cancer and carcinogenesis are complicated and multifactor processes that results in several biochemical alterations in a single cell. All diseases can most likely be attributed to multifactorial effects, and these factors may interact with each other, hence inducing biological changes in a single cell over a period of time. The interaction of the multifactorial effects aggravates the cancer. To solve the extraordinary complexity of cancer and provide excellent therapeutic drugs, several researchers have focused their work on discovering new multitarget drugs which are able to interact with multiple altered pathways [1,2]. In the efforts to find new drugs with these capabilities, scientists have focused on many different characteristics of cancer biology during their research. Among the anticancer drugs discovered recently, various sulfonamides possess potent anticancer properties [3,4] which is attributed to their ability to inhibit carbonic anhydrase enzymes [5]. Carbonic anhydrases is a family of Zn-based metalloenzymes that catalyze the interconversion between carbon dioxide and bicarbonate with generation of protons. The carbonic anhydrase isozyme IX (CA IX) is reported to be associated with tumorigenesis being highly expressed in hypoxic tumors with limited expression in normal tissues [6]. Thiourea derivatives represent one of the most promising classes of anticancer agents with a wide range of activities against various leukemia and solid tumors [7,8,9]. In previous work, combinations of sulfonamide and thiourea derivatives have produced promising anticancer agents [10,11,12]. The importance of this paper lies in that the next generation sulfonamide-thiourea derivatives might be more efficacious as anticancer agents. Since one of the common methods for drug design in medicinal chemistry is varying substituents, we report herein the synthesis and *in vitro* anticancer evaluation of a novel series of thioureido-sulfonamide derivatives as a continuation of our ongoing research project concerned with the synthesis of novel heterocyclic compounds as anticancer agents, where we have identified novel classes of pyrrole, pyrrolopyrimidine, 4-aminopyridine, quinolines and sulfonamides and these new derivatives showed significant anticancer activity on different biological targets and human cancer cell lines [13,14,15,16,17].

## 2. Results

### 2.1. Chemistry

The aim of this work was to design and synthesize a novel series of thioureido-sulfonamide derivatives and examine their potential anticancer activity starting with the reported isothiocyanatobenzenesulfonamide **2** which was prepared by the reaction of sulfanilamide with thiophosgene following a reported method [18].

**Scheme 1:**
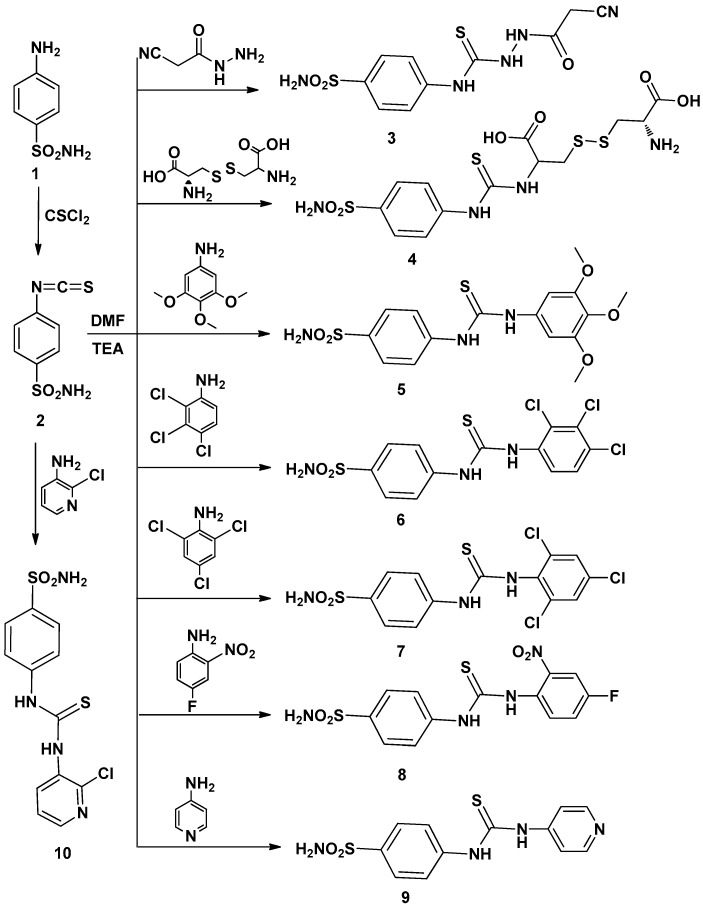
Synthetic pathways for compounds **2**–**10**.

**Scheme 2:**
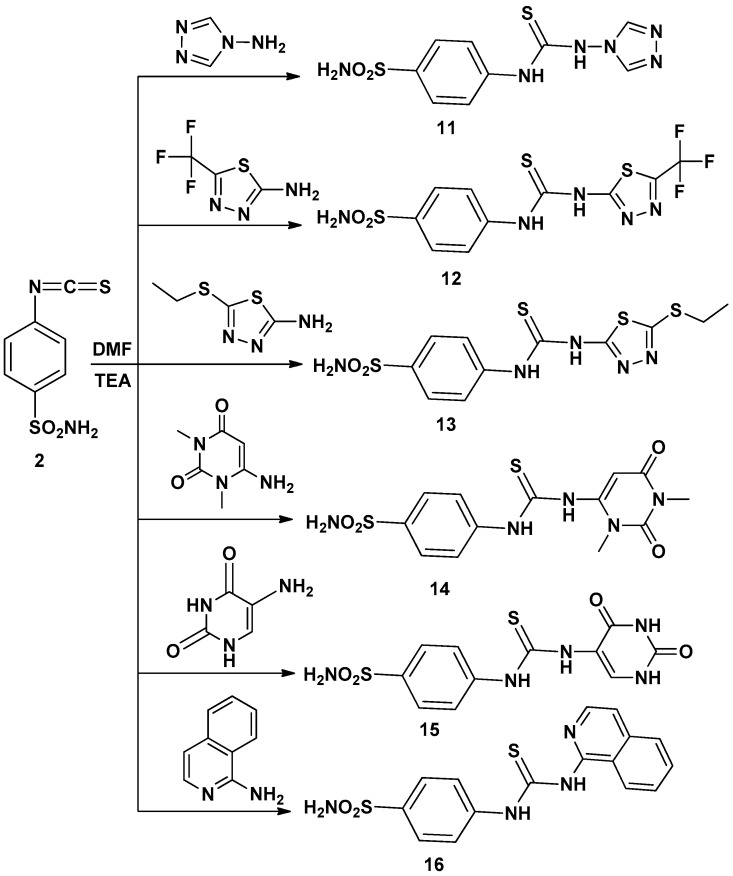
Synthetic pathways for compounds **11**–**16**.

Thus, interaction of compound **2** with several amines in dry DMF containing triethylamine as catalyst afforded the corresponding sulfonamide derivatives **3**–**16** (Scheme 1 and Scheme 2) according to the reported methods [11,12,19]. The structures of the obtained compounds were established on the basis of elemental analyses and spectral data. The IR spectra of compounds **3**–**16** showed the absence of N=C=S groups and presence of absorption bands for (NH), (CH arom.), (CH aliph.), (C=S) and (SO_2_).

The ^1^H-NMR spectra of compounds **3**–**16** exhibited a singlet at 9.3–14.4 ppm assigned to the 2NH groups of thiourea which were exchanged upon deuteration, in addition to the corresponding protons assigned to the introduced aromatic and heterocyclic moieties.

The ^13^C-NMR spectra of compounds **3**–**16** exhibited additional signals for the introduced C=S group of thiourea falling in the range of δ 163.7–188.9 ppm, which is in conformity with the assigned structures. The mass spectra of compound **3**–**16** showed molecular ion peaks at their respective *m/z* values, supported by the elemental analyses data which were found within the limit of 0.4% of theoretical values for all the synthesized compounds.

### 2.2. In-Vitro Anticancer Evaluation

The synthesized compounds were evaluated for their *in vitro* anticancer activity against human lung cancer cell line (A549), cervical (HeLa) cancer cell line, colorectal cell line (LoVo) and breast cancer cell line (MDA-MB-231) using doxorubicin as reference drug. Doxorubicin (CAS, 25316-40-9), an anthracycline intercalating agent, is the reference compound used in this study is a potent anticancer drug. It works by intercalating DNA and thus inhibiting macromolecular biosynthesis. It is mostly used against of a wide range of cancers, including acute leukemia’s Hodgkin’s disease, and other lymphomas and cancers of the breast, adrenal cortex, colorectal, cervical, endometrium, lung, ovary, colon, liver and other sites. By Plotting the relationship between surviving fraction and drug concentration we obtained the survival curve of cancer cell lines and calculated the IC_50_ value, which corresponds to the concentration required for 50% inhibition of cell viability. The results are presented in Table 1, where some compounds exhibit fair activity compared to doxorubicin as reference drug. In case of human lung cancer cell line (A549) compounds **6**, **9**, and **10** were the most potent on this cell line with lower IC_50_ values than doxorubicin ranging from 171.4–272.1 µM. Moreover, the most potent compounds on HeLa cell lines were the sulfonamide derivatives **4**, **6**, **10** and **12** (IC_50_ range 137.5–259.6 µM). In case of the colorectal cell line (LoVo), four compounds (**6**, **7**, **10** and **16**) exhibited better activity than doxorubicin. Mild activity was observed for the synthesized compounds against breast cancer cell line (MDA-MB-231) where, the most potent compounds were compounds **2**, **6**, **7**, **12**, **13** and **16** which are found to be less active than doxorubicin. Generally, the breast cancer cell line (MDA-MB-231) was the most sensitive to the synthesized compounds. Considering broad spectrum anticancer activity, closer examination of the data presented in Table 1, revealed that compound **6** was the most active members of this study, showing effectiveness toward the four cell lines. By examining the SAR of the synthesized compounds, the starting isothiocyanate derivative **2** showed low activity on the tested cell lines, and findings for compounds **3**–**5**, **8**, **9** and **11**–**16** were similar. Meanwhile, introduction of a trichlorobenzene moiety in compound **6** significantly increased the activity against the four cell lines. We also concluded that the 2,3,4-position of chlorine atoms on the benzene ring was better than the 2,4,6-position in compound **7** which showed slightly lower activity towards the tested cell lines. In addition, introduction of a chloropyridine moiety in compound **10** also resulted in high activity from which we can suggest the importance of aryl/heteroaryl chlorine derivatives in these anticancer agents.

## 3. Experimental Section

### 3.1. General Information

Melting points (uncorrected) were determined in open capillary on a Gallen Kamp melting point apparatus (Sanyo Gallen Kamp, Southborough, UK). Precoated silica gel plates (Kieselgel 0.25 mm, 60 F254, Merck, Darmstadt, Germany) were used for thin layer chromatography. A developing solvent system of chloroform/methanol (8:2) was used and the spots were detected by ultraviolet light. IR spectra (KBr disc) were recorded using an FT-IR spectrometer (Perkin Elmer, Norwalk, CT, USA). Mass spectra were recorded on a 600 GC/MS (Clarus, Middletown, CT, USA) and TQ 320 GC/MS/MS mass spectrometers (Varian, West Sussex, UK), ^1^H-NMR spectra were scanned on a NMR spectrometer (Bruker AXS Inc., Flawil, Switzerland), operating at 500 MHz for ^1^H-NMR and 125.76 MHz for ^13^C-NMR. Chemical shifts are expressed in δ-values (ppm) relative to TMS as an internal standard, using DMSO-*d*_6_ as a solvent. Elemental analyses were done on a model 2400 CHNSO analyser (Perkin Elmer, Norwalk, CT, USA). All the values were within ±0.4% of the theoretical values. All reagents used were of AR grade. The starting material 4-chloro-2-phenylquinazoline was purchased from Sigma (St. Louis, MO, USA) and was directly used for the preparation of target compounds.

### 3.2. Chemistry: General Procedure for the Synthesis of Sulfonamide Derivatives ***3**–**16***

A mixture of 4-isothiocyanato benzenesulfonamide **2** (2.14 g, 0.01 mol) and an appropriate amine (0.012 mol) in dry dimethylformamide (15 mL) containing trimethylamine (0.3 mL) was refluxed for 24 h., then left to cool. The solid product formed upon pouring onto ice/water was collected by filtration and recrystallized from ethanol-dimethylformamide to give **3**–**16**, respectively.

*2-(2-Cyanoacetyl)-N-(4-sulfamoylphenyl)hydrazine carbothioamide* (**3**): Yield, 86%; m.p. 153.6 °C. IR (KBr, cm^−1^): 3312, 3214 (NH, NH_2_), 3099 (CH arom.), 2954, 2853 (CH aliph.), 1655 (C꞊O), 1387, 1157 (SO_2_), 1250 (C=S). ^1^H-NMR (DMSO-*d*_6_): 3.3 [s, 2H, CH_2_], 6.4–8.0 [m, 6H, Ar-H + SO_2_NH_2_], 9.3, 10.4, 13.0 [3s, 3NH, exchangeable with D_2_O]. ^13^C-NMR (DMSO-*d*_6_): 25.3, 120.3 (2), 126.1, 127.8 (2), 139.2, 141.5, 152.3, 163.7. MS *m*/*z* (%): 313 (M^+^) (13.44), 156 (100). Anal. Calcd. for C_10_H_11_N_5_O_3_S_2_ (313): C, 38.33; H, 3.54; N, 22.35. Found: C, 38.09; H, 3.19; N, 22.64.

*2-Amino-3-(2-carboxy-2-(3-(4-sulfamoylphenyl)thioureido)ethyl)disulfanyl)propanoic acid* (**4**): Yield, 68%; m.p. 252.6 °C. IR (KBr, cm^−1^): 3354 (OH), 3253, 3185 (NH, NH_2_), 3030 (CH arom.), 2964, 2858 (CH aliph.), 1783 (2C=O), 1388, 1162 (SO_2_). ^1^H-NMR (DMSO-*d*_6_): 3.0, 3.5 [m, 6H, CH-CH_2_-S-S-CH_2_-CH], 7.0–7.8 [m, 6H, Ar-H + SO_2_NH_2_], 8.0 [s, 2H, NH_2_, exchangeable with D_2_O], 10.9 [s, 2H, 2NH, exchangeable with D_2_O], 13.9 [s, 2H, 2OH, exchangeable with D_2_O], ^13^C-NMR (DMSO-*d*_6_): 40.2, 40.3, 66.8 (2), 123.6 (2), 126.7 (2), 140.9, 142.1, 157.3, 168.1, 178.5. MS *m*/*z* (%): 455 (M^+^) (18.48), 152 (100). Anal. Calcd. for C_13_H_18_N_4_O_6_S_4_ (455): C, 34.35; H, 3.99; N, 12.33. Found: C, 34.65; H, 4.33; N, 12.59.

*4-(3-(3,4,5-Trimethoxyphenyl)thioureido)benzenesulfonamide* (**5**): Yield, 78%; m.p. 203.7 °C. IR (KBr, cm^−1^): 3345, 3231, 3155 (NH, NH_2_), 3100 (CH arom.), 2967, 2829 (CH aliph.), 1328, 1180 (SO_2_), 1258 (C=S). ^1^H-NMR (DMSO-*d*_6_): 3.8 [s, 9H, 3OCH_3_], 6.6–7.7 [m, 8H, Ar-H + SO_2_NH_2_], 9.9 [s, 2H, 2NH, exchangeable with D_2_O].^13^C-NMR (DMSO-*d*_6_):56.3 (2), 60.5, 102.2 (2), 123.3 (2), 127.7 (2), 130.5, 135.0, 135.4, 139.6, 153.7 (2), 179.7. MS *m*/*z* (%): 397 (M^+^) (9.23), 229 (100). Anal. Calcd. for C_16_H_19_N_3_O_5_S_2_ (397): C, 48.35; H, 4. 82; N, 10.57. Found: C, 48.71; H, 4.56; N, 10.27.

*4-(3-(2,3,4-Trichlorophenyl)thioureido)benzensulfonamide* (**6**): Yield, 89%; m.p. 231.3 °C. IR (KBr, cm^−1^): 3353, 3245, 3211(NH), 3010 (CH arom.), 1330, 1159 (SO_2_), 1268 (C=S), 725 (C–Cl).^1^H-NMR (DMSO-*d*_6_): 6.6–7.7 [m, 8H, Ar-H + SO_2_NH_2,_], 10.3[s, 2H, 2NH exchangeable with D_2_O]. ^13^C-NMR (DMSO-*d*_6_): 123.0 (2), 126.5, 128.8 (2), 129.5, 130.5, 131.6, 137.6, 139.8, 140.1, 142.6, 180.0. MS *m*/*z* (%): 411 (M^+^) (36.71), 74 (100). Anal. Calcd. for C_13_H_10_Cl_3_N_3_O_2_S_2_ (411): C, 38.02; H, 2.45; N, 10.23. Found: C, 38.32; H, 2.18; N, 10.54.

*4-(3-(2,4,6-Trichlorophenyl)thiouredo)benzenesulfonamide* (**7**): Yield, 76%; m.p. 225.2 °C. IR (KBr, cm^−1^): 3425, 3369, 3327, 3249 (NH, NH_2_), 3080 (CH arom.), 1393, 1181 (SO_2_), 1299 (C=S), 854 (C–Cl).^1^H-NMR (DMSO-*d*_6_):7.3–7.8 [m, 8H, Ar-H + SO_2_NH_2_], 10.3 [s, 2H, 2NH, exchangeable with D_2_O]. ^13^C-NMR (DMSO-*d*_6_): 118.8 (2), 127.9, 128.7 (2), 133.1 (2), 136.2, 139.8, 140.1 (2), 141.0, 181.0. MS *m*/*z* (%): 411 (M^+^) (12.71), 93 (100). Anal. Calcd. for C_13_H_10_Cl_3_N_3_O_2_S_2_ (411): C, 38.02; H, 2.45; N, 10.23. Found: C, 37.82; H, 2.76; N, 9.91.

*4-(3-(4-Fluoro-2-nitrophenyl)thioureido)benzenesulfonamide* (**8**): Yield, 77%; m.p. 206.3 °C. IR (KBr, cm^−1^): 3482, 3359, 3244 (NH, NH_2_), 3105 (CH arom.), 1377, 1182 (SO_2_), 1273 (C=S). ^1^H-NMR (DMSO-*d*_6_): 7.0–7.8 [m, 9H, Ar-H + SO_2_NH_2_], 10.3 [s, 2H, +2NH, exchangeable with D_2_O]. ^13^C-NMR (DMSO-*d*_6_): 110.1, 121.4, 123.2 (2), 125.3, 126.7, 129.1 (2), 139.8, 142.8, 143.9, 153.1, 180.1. MS *m*/*z* (%): 370 (M^+^) (8.76), 139 (100). Anal. Calcd. for C_13_H_11_ FN_4_O_4_S_2_ (370): C, 42.16; H, 2.99; N, 15.13. Found: C, 42.51; H, 2.63; N, 15.41.

*4-(3-Pyridin-4-ylthioureido)benzenesulfonamide* (**9**): Yield, 81%; m.p. 295.6 °C. IR (KBr, cm^−1^): 3411, 3332, 3215 (NH, NH_2_), 3100 (CH arom.), 1590 (C=N), 1332, 1155 (SO_2_), 1245 (C=S). ^1^H-NMR (DMSO-*d*_6_): 6.3–8.3 [m, 10H, Ar-H + SO_2_NH_2_], 10.1 [s, 2H, 2NH, exchangeable with D_2_O].^13^C-NMR (DMSO-*d*_6_): 109.2 (2), 127.1 (2), 127.3 (2), 141.1 (2), 149.7 (2), 159.8, 178.3. MS *m*/*z* (%): 308 (M^+^) (15.18), 77 (100). Anal. Calcd. for C_12_H_12_N_4_O_2_S_2_ (308): C, 46.74; H, 3.92; N, 18.17. Found: C, 46.46; H, 3.64; N, 18.49.

*4-(3-(2-Chloropyridin-3-yl)thioureido)benzenesulfonamide* (**10**): Yield, 74%; m.p. 245.3 °C. IR (KBr, cm^−1^): 3454, 3363, 3243 (NH, NH_2_), 3102 (CH arom.), 1623 (C=N), 1381, 1155 (SO_2_), 1253 (C=S), 794 (C–Cl). ^1^H-NMR (DMSO-*d*_6_): 7.4–8.3 [m, 9H, Ar-H + SO_2_NH_2_], 10.3 [s, 2H, 2NH, exchangeable with D_2_O]. ^13^C-NMR (DMSO-*d*_6_): 117.7 (2), 122.2, 122.9, 127.4 (2), 137.9, 140.0, 141.6, 143.2, 146.2, 182.3. MS *m*/*z* (%): 343 (M^+^) (1.89), 112 (100). Anal. Calcd. for C_12_H_11_ClN_4_O_2_S_2_ (343): C, 42.04; H, 3.23; N, 16.34. Found: C, 42.29; H, 3.53; N, 16.05.

*4-(3-4H-1,2,4-Triazol-4-ylthioureido)benzenesulfonamide* (**11**): Yield, 66%; m.p. 247.8 °C. IR (KBr, cm^−1^): 3346, 33235, 3175 (NH, NH_2_), 3064 (CH arom.), 1593 (C=N), 1407, 1156 (SO_2_), 1262 (C=S). ^1^H-NMR (DMSO-*d*_6_): 7.3–8.8 [m, 8H, Ar-H + SO_2_NH_2_], 10.1 [s, 2H, 2NH, exchangeable with D_2_O]. ^13^C-NMR (DMSO-*d*_6_): 125.2 (2), 127.5 (2), 137.9, 140.4, 142.2, 145.3, 180.6. MS *m*/*z* (%): 298(M^+^) (4.64), 68 (100). Anal. Calcd. for C_9_H_10_N_6_O_2_S_2_ (298): C, 36.23; H, 3.38; N, 28.17. Found: C, 36.51; H, 3.07; N, 27.85.

*4-(3-(5-(Trifluoromethyl)-1,3,4-thiadiazol-2-yl)thioureido)benzenesulfonamide* (**12**): Yield, 59%; m.p. 234.5 °C. IR (KBr, cm^−1^): 3352, 3243, 3185 (NH, NH_2_), 3009 (CH arom.), 1586 (C=N), 1407, 1181 (SO_2_), 1290 (C=S).^1^H-NMR (DMSO-*d*_6_): 7.3–7.9 [m, 6H, Ar-H + SO_2_NH_2_], 10.3 [s, 2H, 2NH, exchangeable with D_2_O]. ^13^C-NMR (DMSO-d_6_): 116.4, 123.3 (2), 126.7 (2), 139.8, 142.8, 151.8, 158.6, 180.1. MS *m*/*z* (%): 383 (M^+^) (14.63), 169 (100). Anal. Calcd. for C_10_H_8_F_3_N_5_O_2_S_3_ (383): C, 31.33; H, 2.10; N, 18.27. Found: C, 31.62; H, 2.42; N, 18.55.

*4-(3-(5-(Ethylthio)-1,3,4-thiadiazol-2-yl)thioureido)benzenesulfonamide* (**13**): Yield, 89%; m.p. 232.2° C. IR (KBr, cm^−1^): 3352, 3220, 3182 (NH, NH_2_), 3078 (CH arom.), 2945, 2853 (CH aliph.), 1595 (C=N), 1402, 1182 (SO_2_), 1243 (C=S). ^1^H-NMR (DMSO-*d*_6_): 1.3 [t, 3H, CH_3_], 3.2 [q, 2H, CH_2_], 7.2–7.9 [m, 6H, Ar-H + SO_2_NH_2_], 10.3, 10.7 [2s, 2H, 2NH, exchangeable with D_2_O]. ^13^C-NMR (DMSO-*d*_6_):15.2, 28.3, 122.2 (2), 127.8 (2), 139.8, 142.8, 154.6, 164.5, 180.0. MS *m*/*z* (%): 376 (M^+^) (17.26), 145 (100). Anal. Calcd. for C_11_H_13_N_5_O_2_S_4_ (376): C, 35.18; H, 3.49; N, 18.65. Found: C, 35.49; H, 3.18; N, 18.36.

*4-(3-(1,3-Dimethyl-2,6-dioxo-1,2,3,6-tetrahydropyrimidin-4-yl)thioureido)benzene-sulfonamide* (**14**): Yield, 90%; m.p. 247.4 °C. IR (KBr, cm^−1^): 3398, 3254, 3225 (NH, NH_2_), 3100 (CH arom.), 2946, 2880 (CH aliph.), 1692 (2C=O), 1381, 1183 (SO_2_), 1235 (C=S).^1^H-NMR (DMSO-*d*_6_): 3.1, 3.2 [2s, 6H, 2NCH_3_], 6.7 [s, 1H, CH], 7.3–7.9 [m, 6H, Ar-H + SO_2_NH_2_], 10.3, 14.4 [2s, 2H, 2NH,exchangeable with D_2_O]. ^13^C-NMR (DMSO-*d*_6_): 27.5, 29.6, 75.3, 123.2 (2), 126.0 (2), 141.4, 142.5, 152.0, 161.9, 163.8, 188.9. MS *m*/*z* (%): 369 (M^+^) (9.28), 138 (100). Anal. Calcd. for C_13_H_15_N_5_O_4_S_2_ (369): C, 42.27; H, 4. 09; N, 18.96. Found: C, 42.51; H, 4.33; N, 18.62.

*4-(3-(2,4-Dioxo-1,2,3,6-tetrahydropyrimidin-5-yl)thioureido)benzenesulfonamide* (**15**): Yield, 67%; m.p. >360 °C. IR (KBr, cm^−1^): 3367, 3286, 3155 (NH, NH_2_), 3013 (CH arom.), 1698, 1671 (2C=O), 1316, 1184 (SO_2_), 1275 (C=S). ^1^H-NMR (DMSO-*d*_6_): 6.8–8.1 [m, 7H, Ar-H + SO_2_NH_2_], 9.3, 10.0, 10.7, 11.0 [4s, 4H, 4NH, exchangeable with D_2_O]. ^13^C-NMR (DMSO-*d*_6_):112.9, 122.4 (2), 126.5 (2), 127.9, 139.7, 142.9, 150.2, 161.9, 162.1, 180.4. MS *m*/*z* (%): 341 (M^+^) (2.77), 111 (100). Anal. Calcd. for C_11_H_11_N_5_O_4_S_2_ (341): C, 38.70; H, 3.25; N, 20.52. Found: C, 38.37; H, 3.56; N, 20.24.

*4-(3-Isoquinolin-1-ylthioureido)benzenesulfonamide* (**16**): Yield, 87%; m.p. 217.1 °C. IR (KBr, cm^−1^): 3313, 3286, 3174 (NH, NH_2_), 3087 (CH arom.), 1635 (C=N), 1397, 1189 (SO_2_), 1213 (C=S). ^1^H-NMR (DMSO-*d*_6_): 7.4–8.8 [m, 10H, Ar-H + SO_2_NH_2_], 10.8, 11.8 [2s, 2H, 2NH, exchangeable with D_2_O].^13^C-NMR (DMSO-*d*_6_): 111.6, 115.2, 119.7 (2), 122.2, 125.1, 126.6, 127.2 (2), 129.4, 135.6, 137.4, 140.4, 141.0, 170.0, 179.7. MS *m*/*z* (%): 358 (M^+^) (47.82), 129 (100). Anal. Calcd. for C_16_H_14_N_4_O_2_S_2_ (358): C, 53.61; H, 3.94; N, 15.63. Found: C, 53.36; H, 3.62; N, 15.36.

### 3.3. In-Vitro Anticancer Evaluation

#### 3.3.1. Cell Culture

Human cancer cell lines HeLa (cervical), A549 (lungs) and LoVo (colorectal) were grown in DMEM + GlutaMax (Invitrogen, Carlsbad, CA, USA), and MDA-MB-231 (breast) were grown in DMEM-F12 + GlutaMax) medium (Invitrogen), supplemented with 10% heat-inactivated bovine serum (Gibco, Gaithersburg, MD, USA) and 1x penicillin-streptomycin (Gibco) at 37 °C in a humified chamber with 5% CO_2_ supply.

#### 3.3.2. Cytotoxicity Assay

The *in vitro* anticancer screening was done at pharmacognosy Department, College of Pharmacy, King Saud University Riyadh Saudi Arabia. Cells were seeded (10^5^ cells/well) in 96-well flat-bottom plates (Becton-Dickinson Labware, Franklin Lakes, NJ, USA) a day before treatment and grown overnight. Compounds were dissolved in dimethyl sulfoxide (DMSO; Sigma) and finally prepared as 1.0 mg/mL stocks, respectively in the culture media. The final concentration of DMSO never exceeded 0.1% in the treatment doses. Six different doses of compounds (400, 200, 100, 50, 25 and 10 µM) were further prepared by diluting the stocks in culture media, and cells were treated (in triplicate/dose). Doxorubicin was included as standard reference drug (positive control) and untreated culture was considered as negative control. The treated cultures were further incubated for 48 h. At 48 h post-treatment, cell viability test was performed using TACS MTT Cell Proliferation and Viability Assay Kit (TACS, Abcam, Cambridge, MA, USA) as per manufacturer’s instructions. The optical density (OD) was recorded at 570 nm in a microplate reader (EL × 800, BioTek, Winooski, VT, USA) and cell survival fraction was determined. The cell survival fraction was calculated as [(A − B)/A], where A and B are the OD of untreated and of treated cells, respectively. The relation between surviving fraction and drug concentration is plotted to get the survival curve of each tumor cell line after the specified time. The concentration required for 50% inhibition of cell viability (IC_50_) was calculated and compared with the reference drug doxorubicin. Compounds that fail to inhibit 50% of cell viability are considered to be inactive. The results are given in Table 1.

## 4. Conclusions

The present work describes the synthesis of a novel series of thioureido-sulfonamide derivatives. 4-(3-(2,3,4-Trichlorophenyl)thioureido)benzensulfonamide (**6**) 4-(3-(2,4,6-trichlorophenyl)-thio-ureido)benzenesulfonamide (**7**) and 4-(3-(2-chloropyridin-3-yl)thioureido)benzenesulfonamide (**10**) were the most potent candidates in the *in vitro* anticancer screening study, showing higher activity than doxorubicin. However, further investigation relating the structure and the activity of the sulfonamide and thiourea derivatives as well as their stability under biological conditions is required. These detailed investigations could be helpful in designing more potent anticancer agents for therapeutic use.

## Figures and Tables

**Table 1:** *In vitro* anticancer screening of the synthesized compounds against four cell lines.

Sample Code	A549 (Lungs)	HeLa (Cervical)	LoVo (Colorectal)	MDA-MB-231 (Breast)
IC_50_(µM) ± SD (*n* = 3)				
2	NA	NA	NA	446.1 ± 0.01
3	NA	NA	NA	NA
4	NA	259.6 ± 0.06	NA	NA
5	NA	Na	NA	NA
6	**171.4 ± 0.32**	**137.5 ± 0.12**	**135.2 ± 0.01**	**121.9 ± 0.11**
7	NA	NA	89.47 ± 0.09	283.9 ± 0.11
8	NA	NA	NA	NA
9	199.5 ± 0.12	NA	NA	NA
10	272.1 ± 0.07	149.3 ± 0.03	305.1 **±** 1.00	NA
11	NA	NA	NA	NA
12	NA	150.9 ± 0.04	NA	163.2 ± 0.06
13	NA	NA	NA	267.3 ± 0.01
14	NA	NA	NA	NA
15	NA	NA	NA	NA
16	NA	NA	301.4 ± 1.00	134.4 ± 0.02
Doxorubicin	**283.5 ± 0.01**	**120.7 ± 0.09**	**374.4 ± 1.00**	**26.5 ± 0.54**

NA = Not Active.
